# Outcomes of lateral approach in robot-assisted radical prostatectomy: insights from a single-surgeon experience

**DOI:** 10.1007/s11701-023-01772-y

**Published:** 2024-01-13

**Authors:** Carlo Giulioni, Daniele Castellani, Nam-Son Vuong, Julien Riviere, Julie Piechaud-Kressmann, Laurent Hugo Lopez, Thierry Piechaud, Jean-Baptiste Roche, Jean Rouffilange, Jean-Luc Hoepffner, Andrea Benedetto Galosi, Richard Pierre Gaston, Grégory Pierquet

**Affiliations:** 1 Department of Urology, Polytechnic University of Marche, Azienda Ospedaliera Universitaria della Marche, 71 Conca Street, 60126 Ancona, Italy; 2 Urology Unit, Clinique Saint Augustin, Bordeaux, France

**Keywords:** Prostate cancer, Robot-assisted radical prostatectomy, Lateral approach, Urinary continence, Erectile function

## Abstract

In the era of robotic prostate surgery, various techniques have been developed to improve functional outcomes. Urinary continence has shown satisfactory results, but the preservation of lateral nerves to the periprostatic capsule is only achievable by sparing the pubovesical complex. This study aims to present the first cases of lateral-approach robot-assisted radical prostatectomy (LRRP) performed by a novice surgeon. We conducted a retrospective analysis of 70 prostate cancer patients who underwent LRRP between October 2019 and September 2021, analyzing the perioperative and functional outcomes. The median operative time and intraoperative blood loss were 102 (92–108) minutes and 150 (130–180) mL, respectively. Five minor postoperative complications were reported, and the median hospital stay was 2 (1–2) days. Eleven positive surgical margins occurred. Potency and urinary continence recovery were achieved in 59 (84%) and 66 (94%) patients, respectively, 12 months after surgery. Our analysis shows that LRRP is a safe and effective procedure for prostate cancer surgery. Continence and potency recovery required a short learning curve, with an acceptable recovery rate even in the initial cases.

**Supplementary Information:**

The online version contains supplementary material available at 10.1007/s11701-023-01772-y.

## Introduction

Prostate cancer is the most common**ly** diagnosed tumor in men worldwide, with the highest incidence in Northern Europe [1]. However, mortality rates do not align with incidence rates, thanks to its early diagnosis and treatment in most cases. For localized disease, radical prostatectomy is the recommended surgical treatment, regardless of the risk of tumor progression [2]. The robot-assisted laparoscopic approach is currently considered a reliable option for both oncological and functional outcomes [2].

According to the literature, robot-assisted laparoscopic radical prostatectomy (RARP) demonstrated better urinary continence [3] and potency rates [4] compared to open and laparoscopic approaches due to the high-definition of surgical plans and the ease of instrument manipulation provided by the robotic system. Although the standard (anterior) approach ensures complete recovery of continence in 96.5% of patients, a quarter of cases still complain of erectile dysfunction five years after surgery [5].

The Retzius-sparing (posterior) approach was proposed to preserve anterior structures, such as the Santorini plexus, endopelvic fascia, and puboprostatic ligaments. After evaluating the first 50 consecutive posterior RARP cases, a progressive improvement in outcomes was observed, resulting in a low rate of positive surgical margins (PSM) and good continence recovery [6]. However, recovery of satisfactory erectile function was reported in no more than 80% of patients one year after surgery.

Microscopic evaluation of non-nerve sparing radical prostatectomy specimens has shown that 20–25% of nerves is primarily located along the ventral circumference of the prostatic capsule [7]. Yet, Tewari et al. have described a tri-zonal neural architecture laterally to the bladder neck and seminal vesicles which includes the proximal neurovascular plate, the neurovascular bundle (NVB), and the accessory neural pathways [8]. Therefore, a lateral approach might preserve tissue integrity to improve postoperative recovery of erectile function.

This study aims to assess the oncological and functional outcomes using a lateral approach in robotic-assisted radical prostatectomy (LRRP).

## Methods

### Data collection

A retrospective review of medical records of all patients who underwent LRRP between October 2019 and July 2021 was conducted. A single experienced robotic surgeon performed all procedures. Patients with a pathological diagnosis of prostate cancer with localized disease were included in this analysis [9].

The following demographic data and tumor characteristics were gathered: age, body mass index (BMI), Charlson comorbidity index (CCI), preoperative total serum prostate-specific antigen (PSA) level, prostate volume, biopsy Gleason score, and D’Amico risk group [10]. Intra- and perioperative data, such as operative time (OT), console time (CT), intraoperative blood loss (IBL), length of stay, postoperative complication within 30 days, specimen Gleason score, PSM, and pathological stage were also collected. Early complications (up to 30-day) were graded according to the Clavien-Dindo classification (CD) [11]. Follow-up visits with PSA measurement were scheduled at 1-, 3-, 6-, and 12 months following surgery. Recovery of full urinary continence was considered when the 24-hour pad weight test was zero [12]. The recovery of erectile function was defined complete in the presence of erections adequate for sexual intercourse with or without the use of a phosphodiesterase type 5 enzyme inhibitor.

Formal ethics committee approval was deemed unnecessary for this type of study in our center because retrospective data collection was obtained for clinical purposes, and all the procedures were performed as part of routine care. The study was conducted following the 1964 Helsinki Declaration and its later amendments. All patients signed an informed consent to gather their anonymized data.

### Surgical technique

LRRP is performed using a four-arm da Vinci robot Xi (Intuitive Surgical, Sunnyvale, CA, USA) with the patient in a 30° Trendelenburg position.

**Fig. 1:**
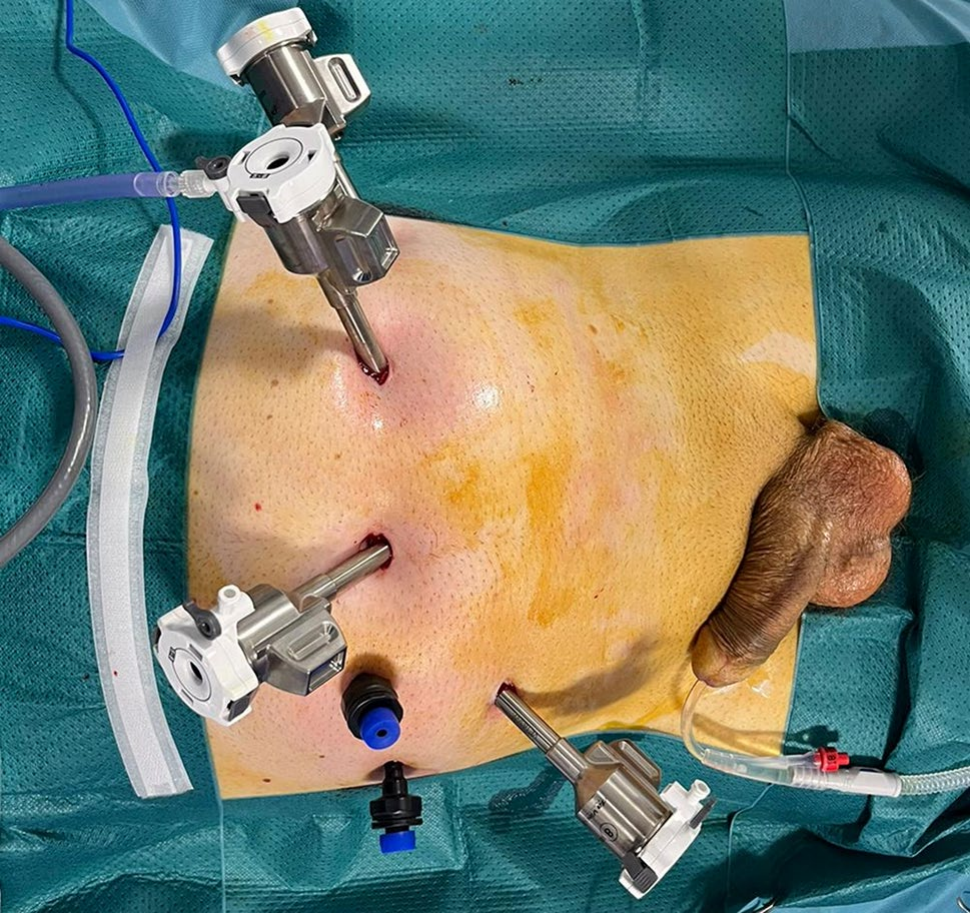
The Location for Port Placement in Lateral Approach in Robot-assisted Radical Prostatectomy

The procedure starts with a sub-umbilical incision and creating pneumoperitoneum using a Veress needle. Trocars are then positioned according to a standard fashion: two robotic trocars on the left umbilical side, a robotic trocar on the right iliac fossa, and two 5-mm assistant trocars on the right umbilical side (Fig. 1). However, the position of the Prograsp and bipolar forceps is reversed to avoid mechanical conflicts during the procedure.

**Fig. 2:**
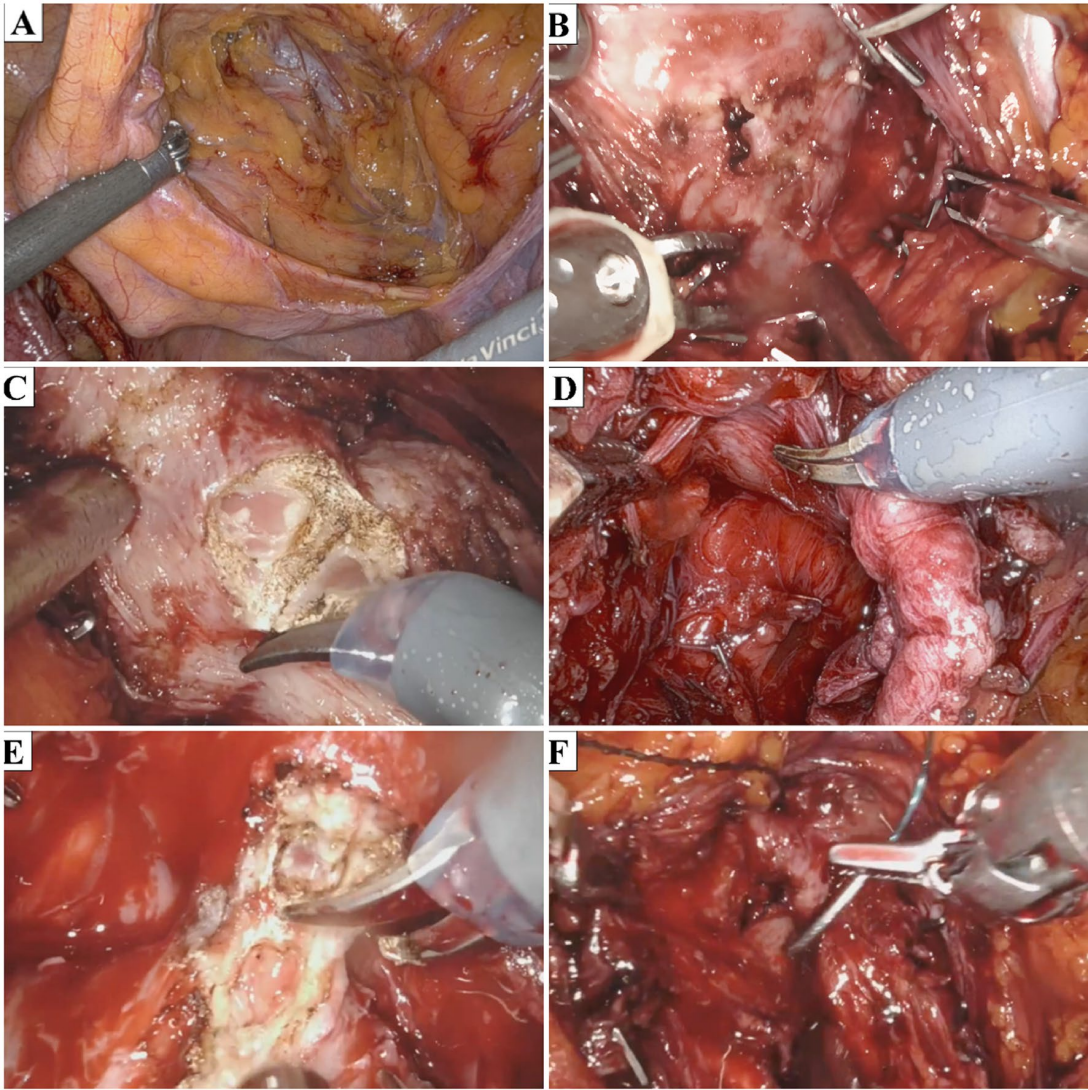
Surgical steps of lateral-approach robot-assisted radical prostatectomy: **a** Peritoneum incision and access to Retzius space; **b** Right intrafascial plane development; **c** Bladder neck incision; **d** Left posterior lateral dissection of the prostate; **e** Urethra section; **f** vesicourethral anastomosis

At beginning, the Retzius space must first be accessed, starting from the right side. A small incision is made in the peritoneum on the right side, starting from the right umbilical artery and continuing until the ipsilateral vas deferens (VD) is reached. Dissection proceeds until the endopelvic fascia, which is incised at the 2 o’clock position to avoid injuring the pericapsular nerve. The right periprostatic fat is then released from the anterior surface of the prostate, and a limited dissection on the left side allows the bladder to descent. Once the right lateral surface of the prostate becomes visible, the Prograsp forceps is used to gently pull the prostate towards the left side (Fig. 2a).

Afterward, dissection carries on the lateral bladder neck until the right seminal vesicle (SV) is reached. Lateral prostatic pedicles are clipped using 5mm titanium clips. When feasible, the NVB is separated from the right lateral surface of the prostate, developing an intrafascial plane (Fig. 2b). The right SV is then isolated laterally, allowing access to the plane between the posterior surface of the prostate and the Denonvillers fascia. The right VD is also cut.

The bladder fibers attached to the edge of the prostate are then peeled and pushed laterally. The bladder neck is fully preserved before being incised, and the vesical catheter is removed (Fig. 2c). The posterior dissection of the prostate continues as much as possible cutting the left SV and VD. No diathermy coagulation is applied close to the NVB, and clips are applied to the left seminal pedicles (Fig. 2d). The apex of the prostate is reached posteriorly.

The dissection of the anterior surface of the prostate continues until the left NVB is released up to the apex from the left side of the prostate. Complete liberation of the prostate is necessary for the section of the urethra (Fig. 2e).

The maximal preservation of the urethra is mandatory to ensure postoperative urinary continence. Once the prostate dissection is finished, a 3-0 V-Loc suture is introduced to carry the tension of the anastomosis. The bladder opening is located on the left side, and a running suture is performed (Fig. 1f).

Pressure on the perineum is applied to expose the urethral side, and the first stitch is placed at 3 o’clock, the second stitch is placed under the first one (at 5 o’clock), and the urethra-vesical anastomosis is completed at the 3 o’clock position. Finally, the anastomosis is tested by filling the bladder, and an ENDOPOUCH RETRIEVER ® bag (Ethicon Inc., Somerville, NJ, USA) is used to remove the specimen. No drain is positioned at the end of the surgery. The procedure can be viewed in the Supplementary video 1.


Supplementary file1 (MP4 335599 KB)

### Statistical analysis

The SPSS software package version 26.0 (IBM Corp., Armonk, NY) was used for all statistical tests. Quantitative variables were reported as median and interquartile ranges, while categorical ones were expressed as absolute frequencies and percentages. T-test and Pearson Chi-square test were performed to compare continuous and categorical variables, respectively. A p value <0.05 was considered statistically significant.

## Results

Overall, the study included 70 patients. Table 1 summarizes the baseline data and demographic characteristics. The median age was 64 (61–68) years, and the median CCI was 6 (5–6,5).

**Table 1:** Baseline data of patients related to Overall group

Variable	Overall (*n*=70)
Age, *years*	64 (61–68)
BMI, *kg/m*^*2*^	26,4 (25,1–28,5)
ASA Score	2(2-3)
Previous surgery for benign prostatic hyperplasia*, n (%)*	
Yes	9 (13)
No	61 (87)
Charlson Comorbidity Index	6 (5–6, 5)
Prostate volume at MRI, *ml*	45 (33–65)
PSA, *ng/ml*	6,75 (5,2 8–8,75)
Gleason score at biopsy*, n (%)*	
≤ 6	13 (18)
7	47 (67)
7	10 (15)
D’Amico risk classification*, n (%)*	
Low	10 (15)
Intermediate	38 (54)
High	22 (31)

Data are presented as medians (interquartile range) and frequencies (proportions). *BMI* Body Mass Index, *ASA* American Society of Anesthesiologists, *MRI* Magnetic resonance imaging, *PSA* Prostate-Specific Antigen

Intraoperative, perioperative and pathology outcomes are reported in Table 2. The median OT, CT, IBL, and length of stay were 102 (92–108) mins, 89 (78–96) mins, 150 (130–180) mL, and 2 (1–2) days, respectively. Five cases of early postoperative complications were reported, and all were CD 1. PSM occurred in 11 cases. Two patients underwent adjuvant radiotherapy due to persistent PSA dosage, and one case of biochemical recurrence occurred 12 months after surgery. No patients died of cancer during the follow-up period. Functional outcomes are reported in Table 3. 81% of patents had full continence within six weeks from surgery, with increasing rates from 3, to 6, and until 12 months after surgery (89%, 91%, and 94%, respectively). One patient required placement of a urethral sling due to persistent stress incontinence. Erections satisfactory for intercourse were reported in 53% of cases at 6-week after surgery. 31 patients required PDE5 Inhibitor. Overall, erectile function rates exhibited a progressive increase, reaching 69% (48/70), 78% (55/70), and 84% (59/70) at 3, 6, and 12 months, respectively.

**Table 2:** Perioperative data of patients related to Overall group

Variable	Overall (*n* = 70)
Operative time, *mins*	102 (92-108)
Console time, *mins*	89 (78-96)
Degree of nerve-sparing, *n (%)*	
Bilateral intrafascial	48 (69)
Unilateral intrafascial	17 (24)
Bilateral interfascial	5 (7)
IOL*, n (%)*	
Yes	17 (24)
No	53 (76)
Intraoperative blood loss,* ml*	150 (130-180)
Clavien-Dindo*, n (%)*	
CD 1	5 (7)
CD 2 or higher	0 (0)
Length of stay*, days*	2 (1–2)
pT*, n (%)*	
pT2	14 (20)
pT3a	39 (56)
pT3b-4	17 (24)
pN*, n (%)*	
pN0	12 (17)
pN+	5 (7)
PSMs*, n (%)*	
Positive	11 (15)
Negative	59 (85)
Overall PSM*, n (%)*	
pT2 PSM	3/11 (25)
pT3 PSM	8/11 (75)

Data are presented as medians (interquartile range) and frequencies (proportions). *IOL* Iliac-obturator lymphadenectomy, *PSM* Positive surgical margin

**Table 3:** Urinary continence and potency rates at single time points for Overall group

Variable	Overall (*n* = 70)
Urinary continence, *n (%)*	
At 6-weeks	57 (81)
At 3-months	62 (89)
At 6-months	64 (91)
At 12-months	66 (94)
Potency, *n (%)*	
At 6-weeks	37 (53)
At 3-months	48 (69)
At 6-months	55 (78)
At 12-months	59 (84)

Data are presented as frequencies (proportions)

## Discussion

Robotic surgery has gained acceptance and spread globally due to its ability to provide enhanced visualization and great precision in hard-to-reach areas. The magnification of the surgical field has allowed for the development of various techniques to preserve the periprostatic structures. Among them, our lateral approach appears helpful as it maximizes the preservation of ultrastructures that support the external urethral sphincter and nerves along the prostatic capsule. Moreover, the application of a single V-loc 3-0 suture demonstrated safety and effectiveness in vesicourethral anastomosis. Hence, it is noteworthy that the barbed suture proved to be non-inferior when compared to a continuous running suture comprising two 3-0 monocryl sutures tied together. This comparison revealed a minimal leakage rate of merely 1.4% within a comprehensive series of 2500 cases, as reported in previous research [13]. This observation aligns with the findings of another study conducted by Zorn et al., further corroborating the comparable security and efficacy of the aforementioned suturing techniques [14].

Despite the aim of preserving periprostatic structures to improve functional outcomes, achieving oncological radicality remains a paramount. The confined spaces may likely increase the incomplete dissection risk during some steps of LRRP. Our study showed a PSM rate of 15%, that is in line with the mean overall rate of 15.2% reported in a review including 16 studies [15]. Six PSM out of 8 PSM were found in patients with pT3 tumors, which are well-known to be associate with a high rate of PSM [16]. Therefore, we argue that LRRP can be considered a safe technique for satisfactory oncological outcomes.

Since the initial RARP description [17], caution was recommended when sparing NVB. Postoperative erectile dysfunction ranges from 14 to 90% [18], with age, preoperative erections, and CCI as the main factors affection erectile function recovery following surgery [19]. Our lateral approach involves a high endopelvic fascia incision to maximize nerve preservation. It is also utilized in select high-risk tumor cases due to lack of correlation between PSM rate and the nerve-sparing technique [20]. The findings of this study reveal a progressive improvement in erectile function recovery over the course of follow-up. In the initial postoperative months, approximately half of the patients received adjunctive medical therapy consisting of PDE5 inhibitors, which facilitate the intracellular accumulation of cGMP within the smooth muscle cells lining blood vessels. Furthermore, the drug’s multifaceted neuroregenerative properties have been validated by animal models, providing substantial evidence to endorse the idea that this pharmaceutical agent not only triggers neurogenesis but also fosters angiogenesis and synaptogenesis within peripheral nerves [21].

The recovery of urinary continence is another key factor to consider when assessing RARP outcomes. In addition to the preservation of the periprostatic tissue, several preoperative risk factors, such as age, preexisting lower urinary tract symptoms, BMI, and membranous urethral length, may also play an important role in functional outcomes [22]. The most well-known approach is the Retzius-sparing RARP, which aims to preserve the anterior support of the prostate. A recent meta-analysis found that the early recovery rate of urinary continence was higher with this technique than with the standard approach (RR = 1.74 and RR = 1.33 after one week and three months from surgery, respectively), although no difference was observed at 12 months (RR = 1.01) [23]. Recently, Ficarra et al. introduced an innovative urethral fixation technique involving a single suture securing the urethral wall to the medial dorsal raphe, positioned within the medial portion of the levator ani muscle, with a subsequent incision of the anterior wall of the urethra, and it is aimed at maintaining the urethral stump in its anatomically correct position [24]. This technique resulted in early recovery of urinary continence in approximately two-thirds of cases (68.6%).

Our technique involves anterior dissection of the prostate sparing the pubovesical ligaments and preserving the structures supporting the external urethral sphincter muscle and the original position of the urethra [25]. Additionally, our accurate intrafascial dissection of the prostate probably contributes to the recovery of urinary continence. A study by Kim et al. found that bilateral nerve-sparing RARP was independently associated with a 1-year postoperative continence return (OR = 3.671) [26]. Most of our patients regained continence after six weeks (81%), and 94% of the at 12-month. Therefore, our technique seems to be promising for gaining a full recovery of continence.

### Limitations of the study

This study has some limitations. First, a significant constraint lies in its retrospective design. The absence of a comparative group hinders our ability to discern the impact of the intervention in question relative to standard RARP approaches.

Secondly, all procedures were performed by a single experienced surgeon; therefore, less skilled surgeons may not be able to achieve the same results particularly before completing their learning curve. Furthermore, the study’s results must be interpreted within the context of a limited sample size. The limited sample size presents a significant impediment in ascertaining after how many cases good outcomes in both oncological and functional outcomes can be achieved.

Consequently, it is recommended that a multicenter study should be performed to validate the findings presented in the present research.

## Conclusion

Our study shows that our technique was feasible in experienced hands and associated with a low rate of early complications and PSM. Our lateral approach demonstrated similar rates of continence recovery compared to other techniques while showing promising results in erection recovery. These results suggest that LRRP can lead to satisfactory oncological and functional outcomes, provided that the surgeon skilled in the standard technique can adopt this approach to improve tissue integrity.

## Data Availability

The datasets used and analyzed during this study are available from the corresponding author upon reasonable request.
